# The psychological impact of the secondary school transition on families of autistic children

**DOI:** 10.1080/20473869.2023.2170004

**Published:** 2023-02-06

**Authors:** L. Yates, S. Keville, A. Ludlow

**Affiliations:** Department of Psychology, Sport and Geography, University of Hertfordshire, Hatfield, UK

**Keywords:** autism, transition, education, psychological distress, mothers

## Abstract

The transition from primary to secondary school is a stressful period for autistic individuals. However, less is known about parental experiences of the school transition, and its impact on the family. This study explored mothers’ perspectives on the psychological impact of the transition to secondary school for their autistic children and their families. Using Interpretative Phenomenological Analysis, semi-structured interviews were analysed to explore the experiences of eight mothers of autistic children at the end of their child’s first year in secondary school. The analysis revealed two superordinate themes: lack of available support and detrimental psychological impact on the family. Mothers reported the negative impact the transition had on themselves, their child, and the wider family. The importance of pre- and ongoing transition support was highlighted to reduce the concerns of children and their parents throughout the transition process. The findings highlighted the need for autism-specific individualized guidance, as well as considering the potential for transition issues to impact on siblings.

In the United Kingdom (UK), at age 11 most children transition from primary to secondary school (Le Métais 2003). As a landmark event it brings considerable change for any child (Lohaus *et al.* 2004). For example, whilst many children report excitement about starting a new school and meeting new friends, they also describe the transition as challenging and anxiety-provoking (Jindal-Snape and Miller 2008). Consequently, during this period children may also experience declines in self-esteem, enjoyment, and academic attainment (Wigfield *et al.* 1991). This can be testing for any child, but for a young person with a diagnosis of autism, where transitions are a distinguishing area of difficulty (American Psychiatric Association (APA 2013), the challenges are likely to be more complex (Mandy *et al.* 2016).

In the UK, a high proportion of children diagnosed with autism(72%) are educated in mainstream settings (Department for Education 2018). Reflecting a movement towards inclusive schooling, this rate has been steadily increasing, enabling children with Special Educational Needs (SEN) to access education in mainstream contexts (United Nations Educational, Scientific and Cultural Organization 1994). However, features of autism, such as sensory sensitivities and communication difficulties, are likely to complicate education experiences, including the transition from primary to secondary school, with secondary schools frequently having over three times as many pupils (Department for Education 2022). Given minor changes in environments can result in confusion and distress for autistic individuals (Groden *et al.* 1994), the louder chaotic physical environment of schools can be overwhelming (Makin *et al.* 2017). Furthermore, in primary schools one main class teacher is often the anchor point with expertise at the individualised teacher/pupil level, yet, at secondary school students are taught by several subject-specialist teachers, each having limited contact time with students (Dillon and Underwood 2012). Therefore, students must adapt and cope with new, differing environments and routines, contributing to the secondary school transition being a challenging time for autistic young people (Mandy *et al.* 2016). Indeed, despite parents’ main priority for the secondary school transition being their child’s well-being, they rated their child’s transition as *extremely difficult* with anxiety/stress considered the main difficulty for their child (Nuske *et al.* 2019).

More positive transition experiences for autistic students have been found in relation to school and student characteristics, the nature of support and also social experiences (Dann 2011). For example, schools that have centred the transition process around autistic children’s own interests and leisure activities were found to engage the children more (Hoy *et al.* 2018). More negative transition experiences, including social isolation, bullying and anxiety, were more commonly associated with an autism diagnosis (Humphrey and Lewis 2008; Carrington and Graham 2001); as well as the lack of available support from the school (Tehee *et al.* 2009), school systems being too rigid in their responses, and poor communication (Podvey *et al.* 2013). There has also been a reported mismatch in the perceptions of key stakeholders regarding available support when transitioning between mainstream schools, with teachers more likely to report the availability of support than parents of children with autism (Bruck *et al.* 2022).

The experience of the school transition can also be challenging for parents of autistic children; with most studies largely utilising parental perspectives to understand their child’s experiences of the transition (Hannah and Topping 2013, Peters and Brooks 2016, Dillon and Underwood 2012). For example, Cremin et al. (2017) interviewed a small group of parents on their experiences of the transition in the Republic of Ireland. Parents recommendations for improvements included more practical support and communication during the transition period, as well as the need to support autistic children in making social attainments and connections within mainstream secondary schools. A similar study in France assessing the experience of the transition over the first year of secondary school found facilitators of the transition to be individualised transition planning, timely attributions of school places and teaching assistants, and early and inclusive orientation meetings; barriers related to a lack of communication or administrative procedures, including unclear expectations by both teachers and parents, lack of information and late decisions (Richter *et al.* 2020).

Studies have found parents reported heightened anxiety during this transition period, stemming from concerns their child would be unhappy at their new school, and the transition would not progress smoothly; support from teachers/services, alongside planning and preparation were considered of highest importance for transition success (Tso and Strnadová 2017). Yet, despite parental anxieties around their child’s ability to manage the social and academic demands of secondary education, parents felt the support for their child during the transition had been inadequate (Tobin *et al.* 2012). Furthermore, successful transition was not predicted by individual child-level factors, such as anxiety or autism symptomology, it was predominately related to higher school- and system-level factors, including lack of preparedness from the primary school, and communication between schools (Makin *et al.* 2017).

While studies exploring parents’ experiences agree there is a lack of available support for parents resulting in heightened feelings of anxiety and isolation (Tso and Strnadová, 2017), there is still limited evidence for the transition’s impact on other aspects of parents’ lives, such as their relationships, career choices, and day-to-day functioning (Tobin *et al.* 2012). Equally, while research has emphasised the importance of the home-school collaboration in facilitating the process (Makin *et al.* 2017, Cremin *et al.* 2017), research now needs to build on how to implement more specific autism provision and support.

Examining parents’ experiences of transition to school and its impact on the rest of the family is important for several reasons. First, the associations are well established between raising an autistic child and parental stress, influencing physical and mental health problems (Karst and Van Hecke 2012). Second, periods of developmental transition can exacerbate or worsen behavioural and emotional issues for autistic children, likely exacerbating strain on caregivers (Cheak-Zamora *et al.* 2015). Indeed, anxiety can manifest via disruptive behaviours in autistic children, such as increases in aggression, self-injurious behaviour and property destruction, and is more prevalent in school-based and community settings (Adams *et al.* 2019).Third, the school transition is likely to have a wider impact on the family, outside of any direct effects on the children themselves (Hetherington *et al.* 2010).

The current research aimed to gain an in-depth understanding of parental experiences on the impact of secondary school transition on themselves and their family. There was a focus on the support parents received to identify provision that made a positive difference. To our knowledge this is the first study to explore richly and directly the lived experience of the impact of this transition on the families of autistic young people in the UK.

## Materials and methods

### Research design

Interpretative Phenomenological Analysis (IPA) was chosen as it draws on the principles of ideography and is concerned with producing comprehensive in-depth analyses of participants’ experiences; IPA guidelines were followed throughout (Smith *et al.* 2009), as were standards for ensuring quality within qualitative research (Nizza *et al.* 2021).

### Participants

Participants were a self-selected sample of 8 mothers accessed and recruited pre-COVID-19 lockdown through the Communication and Autism Team (CAT). Despite the study being advertised to all parents only mothers responded, all with a child with a formal diagnosis of autism, with ages ranging from 11 to 12 years. Participants’ ages ranged from 40 to 48 years (Mean = 45 years, SD = 5.9 years). All children had transitioned from a mainstream primary school and were in the summer term of their first year of mainstream secondary school within one local educational authority in south-east England. One child was female and was the only one to receive an autism diagnosis after the school transition. Five children had a diagnosed co-occurring disorder (see Table 1).

**Table 1.** Participant/child demographic information.

Mother	Age	Ethnicity	Occupation	Child’s diagnosis (including co-occurring disorders)	Gender of child	Age of child	Age of child at diagnosis	Number of siblings *(Age respective to child with autism)*
Anna	45	White British	Nurse/Counsellor	Autism, specific learning difficulty, verbal Dyspraxia	Male	11	Unknown	2 *(1 older, 1 younger)*
Juliet	40	Pakistani	Housewife	Asperger’s syndrome, Dyspraxia, Dyslexia	Male	12	9	2 *(1 older, 1 younger)*
Francesca	48	Hindu/Indian	Pharmacist	Asperger’s syndrome	Male	11	10	1 *(older)*
Louise	44	White British	Retired	Autism, Hyperacusis, Tinnitus	Male	12	9	2 *(older)*
Mary	45	White British	General Practitioner	Asperger’s syndrome	Male	12	8	1 *(younger)*
Becky	37	White British	Receptionist	Autism, Attention Deficit Hyperactivity Disorder, Dyspraxia	Male	12	4	0
Sally	57	White British	Part-time Nurse	Asperger’s syndrome	Female	12	12	2 *(older)*
Diane	45	White British	Nurse	Autism, Selective Mutism	Male	12	8	2 *(older)*

### Interview guide

To facilitate a better understanding of the transition process and the appropriate terminology, the first author attended a CAT transition support group for parents of autistic children. No parent attending this session was involved in the final study. After reviewing existing literature, a semi-structured interview schedule was developed and piloted with two parents not included in the final analysis. Based on feedback from the parents, two questions were reworded. Core questions included:

- Can you tell me about your experience of your child’s transition from primary to secondary school?
- What kind of impact did the secondary transition have on you and your family?
- What types of support did you/your child receive during the transition?
- Is there anything more that could have been done to help you?

### Data collection

The first author conducted all interviews face to face pre-COVID-19 pandemic, at home or in private venues; interviews lasted between 30–50 minutes. Following the interview, participants were given the opportunity to share further information, thanked for their time and were given a debrief sheet which included supportive websites.

### Ethical considerations

Approval was given by the institution’s Ethics Committee (protocol number: LMS/PGT/UH/02826). Researchers ensured confidentiality throughout the study. Participant information sheets were given, and informed consent sought from the participants who were made aware of interviews being audio-recorded, stored, transcribed verbatim with identifying details removed, recordings deleted on transcription, and anonymised data used in a publication.

### Data analysis

IPA was used to interpret the participants’ subjective experience (Smith *et al.* 2009). Transcripts were thoroughly read by the first author to allow familiarisation, followed by a synergetic process noting emerging themes and preliminary interpretations to highlight interesting or significant pieces of the data. Transcripts were re-read and themes capturing the essences of the data were refined. This was systematically repeated with each transcript for a case-by-case analysis. A reviewing and refining process was conducted to ensure emerging themes worked in relation to each other, and the original dataset. Emerging themes were collated across transcripts and analysed for similarities and divergencies, with superordinate and subordinate themes developed across the data. To refine the themes reflexive discussions were conducted throughout the analysis with the third author. To ensure rigour and credibility themes were checked against the transcripts to ensure participants words were represented, with the final themes table and supporting quotes developed with the third author; all quotes within the paper where then agreed by all authors. The finalised table was shared with all participants; with all agreeing the final themes presented in this paper (Nizza *et al.* 2021).

## Results

Analysis of eight semi-structured interviews resulted in the following subordinate and superordinate themes (Table 2).

**Table 2.** Superordinate and subordinate themes.

Superordinate theme	Subordinate themes
Lack of appropriate support	A lonely battle Empty promises Provision specific to supporting parents Unique needs of the individual Lack of awareness surrounding autism
Detrimental psychological impact on the family	Increase in child’s behavioural issues Breakdown in the family unit The emotional impact on the mother

### Lack of available support

This theme explored mothers’ experiences of available support from schools, and the role schools played in their child’s transition. Although discussed separately, their own experiences were inextricably linked to the way the transition personally impacted on their child.

#### A lonely battle

Six mothers used terms such as ‘fight’, representing a warlike battle experience and, with this, their activist attitudes towards getting their child’s needs met. Being advocates, Juliet, Louise, Mary and Sally fought endlessly for their children to ensure their voices were heard, pushing the school to be more proactive in implementing transition support: ‘It was very much my own efforts. They weren’t proactive at all. So, I just kept bugging them until I saw things happening’ (Mary); and ‘I just kept fighting. I kept fighting for what I needed…I can’t cry. I’ve got to fight. I did my crying…no, now it’s time to fight’ (Becky).

Becky and Louise both had to go through a tribunal and ‘appeal to get into the school that could provide the best support’ (Louise); echoing this Anna described the process of a continued fight to keep her son in their preferred school; with no sense of ‘choice’ in the matter, there was a feeling of defeat and powerlessness: ‘I would like him to stay there but they want us to send him to a special school […] I feel I don’t have a choice’.

Getting schools to implement and ‘actually follow through on the plans’ (Becky) was a recurring battle for several of the mothers (Francesca, Mary, Becky, Anna, Louise, Sally). For example, Mary described a battle getting ‘EHC [Education, Health and Care] Plans and transition packages’ in place; and Anna said: ‘transitioning earlier would have helped’ her autistic son, but the process was ‘completely held up’ due to ‘waiting on county who were doing the EHCP’.

For most mothers, there were battles around smaller, yet still significant aspects of settling their autistic child in school, such as practicalities and logistics. For example, part of Juliet’s fight was for her son to be able to have ‘his special pen and cushion’. Mary fought for ‘the basics’, including being given information about her son’s timetable and teachers prior to him starting, yet highlighting how her son only got ‘things like the summer school week because [she] pushed for it and kept contacting the school saying, “what’s on offer?”’.

Sally stated she had to do ‘a lot of [her] own research and work to find out more and get answers…otherwise there isn’t really anyone telling us’. Following a drop in her daughter’s school attendance, she described ‘having to come up with [her] own solutions’ to get her back into school. She had to ‘keep ringing up and asking several people’ for her daughter to be able to access learning support as ‘they were trying to stop her going to learning support when she was having a meltdown’.

Mothers described many small, yet significant, unknown aspects within the new schooling routines which were beyond their control, evoking their own sense of disorientation. Indeed, Juliet wanted guidance on how to support her son with the increased academic pressures, including around ‘his memory, how to revise, how to make notes’; yet, directly stated that she felt ‘lost’ with no-one‘pointing [her] in the right direction’. Consequently, since the transition she expressed being ‘worried about the academic side as he’s [her son] not moving along’. Further, Francesca ‘did not know who to go to’ for extra academic and wellbeing support for her son, which they were ‘not getting from the school’.

The futility of the fight was emphasised when Anna described herself ‘hitting up against a brick wall’ with schools presenting a deliberately unmoveable opposition, where, initially, her child was not offered a place before reluctantly accepting him: ‘The secondary school kept telling us, “We don’t bend”…and I think again it was this, “Let’s put up a barrier to put people off”’.

In contrast, Francesca spoke gratefully about school-based transition support for her son; yet equally, she felt it was a ‘shame’ this was not the case for others. Despite this, she noted a positive outcome was a consequence of her own efforts and representation which seemingly averted a battle: ‘the primary school would have done nothing had I not been to the independent legal body that supports us. The school, no school would have done anything. He wouldn’t have had a diagnosis; we wouldn’t have been to CAMHS’. Perhaps in the midst of this battle and associated fatigue, three mothers mentioned the desire ‘to have someone alongside them’ (Anna) to accompany them through their lonely battle and ‘join up the dots’ (Anna).

Consequently, given Louise’s child had been ‘caught in the middle’ as the ‘primary school felt the onus was on the secondary school…but the secondary school felt the onus was on the primary school’, she felt it was important to identify who was taking ‘responsibility for the transition’.

#### Empty promises

Promised transition procedures never materialised for four mothers (Becky, Francesca, Mary and Sally). Sally stated: ‘we met with the transition people, and we’re going to do this, and this with them, but actually it didn’t work that way at all’; indeed, sometimes there were unhelpful negative consequences from this. For example, Sally recounted a dialogue her daughter had with a class teacher:

…she has had meltdowns in the class, in front of the teachers … and she is often not felt that she is listened to and that increases anxiety. So … now she can leave the class if she wants, but if she puts her hand up and says, “Can I leave? Can I go out?” … “No you can’t go out”. So she’ll get up and walk out. “You’re on detention.”

Further, Becky described ‘a lot of empty promises and a lot of let-downs’ as the school ‘promised the world, but it didn’t happen’. For Becky’s son, the unfilled promises included him visiting the school and having photos of classrooms and teachers prior to starting, and not providing the agreed one-to-one support during unstructured times, as detailed in the Education Health Care Plan (EHCP). This resulted in ‘pulling him out after two weeks’, he was then ‘out of school for six months’ before transitioning to a different secondary school; by constantly ‘starting again’ the journey seemed perpetual with no tangible progress made. Becky despondently concluded:

All the hard work and the preparation and planning was done. All they had to do was follow through on it and implement it. But they didn’t. It was all a big, wasted effort.

Similarly, Sally emphatically encapsulated the futility of this battleground when she stated she ‘didn’t feel [she] could move forward, ever’.

Francesca had ‘done a lot of work with the primary school and the ed psych [educational psychologist] … all the recommendations were made and discussed with the learning support […] none of that was picked up when we transitioned’. She believed the empty promises were reflective of a bigger societal picture, specifically mentioning ‘political agendas’. She felt the issues emerged beyond the teachers with the ‘time constraints, money constraints’ of the ‘wider network impacting on what they can do’. This was reiterated by Mary, who felt the school never intended to fulfil their promises, instead terminology gave the illusion they were meeting their statutory responsibilities: ‘They use these vague terms…but no one actually does anything. It’s all just jargon on paper…said what they needed to say really, tick the boxes'. Similarly, Mary’s son did not get any additional support beyond that of the rest of the class despite being ‘always promised a big handover but didn’t get it’.

Ironically, for Anna, the only promise the school managed to fulfil was her worst fear: ‘they would be pushing to kick him out of the school by the end of year 7′; as with Becky she had to face the daunting prospect of going through another transition to a different school.

#### Provision specific to supporting parents

All mothers’ accounts emphasised the necessity of parental input in guiding the transition plan; for differing reasons due to negative transition experiences previously noted, or positive ones. For example, more positively and following transition, Juliet felt the one-to-one meetings with the head of the department provided ‘a lot of peace of mind’. Indeed, directly supporting their child indirectly supported parents, for example, Diane spoke positively about the transition being ‘well managed’ as the ‘SENCO [SEN coordinator] at the junior school had already written a report and liaised with the special needs team at secondary’. Likewise, Anna valued having someone from the primary school at transition meetings ‘advocating’ for her son, whilst Juliet thought having a teacher from secondary school visit her son as primary school was ‘one of the best things to do’ so he had a ‘familiar face when he started’.

In contrast, Louise’s experience of being passed from person to person, made her recognise the vitalness of ‘a point of contact…someone who works full time’. Diane would have appreciated better post-transition communication with the school, for regular feedback: ‘Some support afterwards and some continuity would have definitely made a difference…a bit more communication about how things are going’.

Timing of support was also highlighted as important, with two mothers frustrated by support deemed inaccessible or inappropriate, with Diane stating: ‘schools are not designed for parents that work’ having to ‘use up a lot of annual leave’ to be able to co-ordinate ‘all the activities, all the meetings’. Louise thought ‘not calling the parents in during the school day’ would ‘make a big difference’ as it also disrupted her son’s daily school routine triggering catastrophic worries for him: ‘They see you, and they’re like, “What are you doing here? Have I got to come home now? Who died?”’. Consequently, recommending alternative proactive pre-transition approaches, Mary noted she would have valued more ‘direct communication between someone who actually knew [her son] at juniors to someone who was really going to be dealing with him in seniors’.

Seeking support elsewhere was vital for mothers to manage the impact from their child’s transition, with Mary, Sally and Juliet stating that parent support groups reduced feelings of isolation, offering a mutual understanding of circumstances: ‘These classes are vital. They are really important…because I didn’t feel alone anymore’ (Juliet). Four mothers believed offering this through school would help consolidate the link between schools and parents during the transition, such that this could be ‘facilitated by somebody who works in the school’ (Louise). Louise also suggested that having ‘a transition pack’ to work through with her child to prepare them for the transition, including ‘how to ask for lunch’ would help her; and Diane shared her positive experience of her son’s school running a ‘transition group, to be introduced gradually into the school, to ask questions, and to teach practical things like how to do your tie up’.

#### The unique needs of the individual

All the accounts reflected the value mothers placed on schools recognising, accepting and catering for their child as individuals, beyond their autism diagnosis, yet six mothers (Diane, Louise, Becky, Sally, Anna, Juliet) felt their maternal child-focussed advice went unheard and their concerns dismissed. For example, Diane expressed frustration when teachers said her son, who also had selective mutism: ‘…would do much better if he joined in and spoke’. She had to point out that the school needed to find an alternative way to accommodate him, given the selective mutism would predominate. In contrast, Francesca believed important pre-transition work ensured the school understood, and her son was prepared: ‘I would use the same model this school has…If that could be introduced to other schools, at a National level, then I think as a parent I would be happy with that’; and Mary described a positive post transition experience: ‘They’ve been brilliantly responsive to meeting with me and talking with me ‘til we get the hang of it’. That said, sometimes, whilst appropriate adaptations were appreciated, such as allocation of an understanding key worker, this was ‘by chance, rather than design’ (Diane).

All mothers recognised the importance of smaller details for autistic children, which ‘made a difference’ (Louise). For example, Louise noted the importance of familiarisation of environmental factors: ‘where is the child’s locker…making them familiar with how to queue up for lunch’. Understanding their child’s idiosyncrasies and unique needs seemed crucial especially as: ‘most [teachers]…didn’t even know he had autism’ (Louise). Perhaps to counter such experiences, especially as parents were unable to do this, Sally suggested good practice could involve teachers: ‘go into the class and observe the child to see what they’re coping with and what they’re not coping with’, as well as paying more attention to their child’s achievements as ‘they didn’t give enough positivity’.

Complicating matters, sometimes adjustments drew attention to their child which made them reluctant to have them: ‘didn’t wanna be the different one…didn’t wanna go to it…and didn’t want a teacher sitting next to him’ (Juliet). Sometimes there was a sense that this may have been due to feeling misunderstood, as Sally recounted her daughter articulated about the school’s management of her anxieties: ‘“…I don’t want to go because they already have an idea in their head of what they want me to do and how they want me to be, and that’s not helping me”’.

#### Lack of awareness surrounding autism

All parents felt that there needed to be more understanding from the teachers on how their child’s autism affected their child’s behaviour: ‘I think not many of them really understand his condition' (Becky). For some parents it was simply improving communication in the school that their child was autistic. For example, Louise highlighted: ‘most of the teachers didn’t even realise he had autism until March, despite us being in there [school] multiple times’. This was further reiterated by Sally:

Initially, the teachers were not aware she was autistic, they weren’t made aware…There have been lots of incidences with the teachers, “[child] has been rude today, she said this and this.” As soon as you say to them, “Are you aware she’s on the spectrum?” … Oh … I … “. And then there hasn’t been another phone call. (Sally)

For others it was the need for teachers to learn how autism impacted on their behaviour and how to manage this. For example, Francesca highlighted the sensory needs of her child impacting the transition to secondary school: ‘The writing went down, everything went down. He couldn’t focus, he couldn’t concentrate, he couldn’t … sensory overload. He struggled with how busy and chaotic it was’.

Whereas some parents felt teachers lost the opportunity to learn about how their child’s autism was affecting their behaviour:

There’s a lot of mind frames you must battle as well, because with, with Asperger’s especially, because when you look at him and you meet him, you’d probably say to me “what you on about?” … But, you know, ‘cos he’s very articulate the way he talks, but obviously if you spent a couple of hours with him … the specialists knew straight away. (Juliet)

Anna recalled: ‘…they said, “we don’t manage children like that, we are not used to children walking out of lessons in this school”’. Whereas Louise suggested: ‘the attitude is very much, “Well we’ve got children in here who are deaf, and they manage alright.” We’ve heard that quite a lot’.

Louise mentioned the importance of tailoring current teachers training away from a ‘one-size-fits-all’ policy for everyone with autism: ‘you can have training on autism, but each child is different. You have to learn about each child’. With Diane highlighting how positive the experience of education can be when that level of autism understanding is present: ‘When he’s had teachers that have understood how to manage autism, and how to manage [child’s name], because obviously autism is different for every child, … he’s thrived and he’s absolutely achieved his best in everything’.

### Detrimental psychological impact on the family

Through discussing transition experiences for their child, all mothers highlighted the negative psychological impact transition had on them and their families.

#### Increase in child’s behavioural issues

All mothers talked about the detrimental impact the school transition had on their child’s behaviour, including increased ‘aggression’ (Anna), ‘swearing…toilet issues’ (Juliet), ‘anger’ (Francesca), and decreased participation in activities, with ‘shying away from things’ (Francesca). Francesca, Mary, Sally, Diane, and Louise noticed shifts in behaviour were especially exhibited in the safe environment of the home: ‘He won’t talk for two hours, or he’s very very angry and he can be quite aggressive…He sorts of holds himself all day…then he just lets it all go at home’ (Louise).

Moreover, the transition and experiences within the new school impacted on their child’s mental health; for example, due to being ‘severely bullied’ Becky’s son left the school, she stated her son ‘got very depressed’ during the time he was out of school. Five mothers noted increased anxiety in their child during the transition. For example, Juliet’s son had panic attacks, which ‘he never had at junior school’; due to the harsh nature of the school environment, Louise’s son tinnitus triggered anxiety which ‘has really got worse since he’s been at secondary school’; for Diane and Louise’s sons, the anxiety manifested as selective mutism which was immediate for Louise’s son: ‘the first two months he didn’t speak’ (Louise); and for Diane the impact came later:

The first six weeks of school were great, and we were surprised at how well he’d adjusted and coped, but then he went back after the October half-term and it was like a different child…I think it was a little bit of a false high…He plummeted to rock bottom.

Consequently, Diane emphasized the importance of schools recognising the transition as a longer-term process rather than ‘a done and dusted deal by week two’.

#### Breakdown in the family unit

Their child’s transition was also a significant time of change for the whole family, having to make physical adaptations which impacted on the family’s emotional well-being. Louise noted: ‘We’ve all had to adapt in ways we didn’t realise we would…We’ve had to make a lot of changes since he’s been at secondary school’.

Six mothers (Anna, Juliet, Francesca, Louise, Sally, Diane) referred to shifts in sibling relationships. For example, Anna spoke of the ‘massive impact’ the transition had on her other children, as one son had come to ‘hate him [her transitioning son] now, as he can’t cope with the stress’. Juliet reflected similar feelings with her two sons ‘finding it harder together, because he [her autistic son] was getting more tired. She explained the eldest ‘doesn’t really understand [his] issues and problems’ and ‘is very embarrassed with him’ now ‘they’re in the same school’. With familial issues emerging, Sally felt she took on the role of ‘mediating… all the time’, adding another pressure into her life.

The transition fractured the family unit, with Louise and Sally noting their other children avoided coming home; or ‘tending to go to their own room…it’s unusual to have [them] all together as a family’ (Sally). This seemed emotionally difficult for both mothers, with Sally remarking that home: ‘often it isn’t a nice atmosphere’. For Louise, the new school routines meant less time to spend with her other children, impacting her own wellbeing: ‘you’ve not spent any time together, and as a mother, I feel guilty about that’.

The school transition impacted beyond their parenting role, to the marital relationship. Due to many disturbed nights comforting her son about his transition-based anxieties and tinnitus, Louise described home as a ‘madhouse’ with having to ‘put a bed downstairs to the front room which [her] husband sleeps on quite often’. Anna almost dismissed the idea there was any marital relationship left, they were just trying to survive:

My husband and I, God knows about the relationship, as it’s about functioning. It’s about getting up, getting the children to school, and…and it’s about functioning, and surviving. That’s what it’s all about…

There was an almost wistful quality with Anna pausing before adding ‘whereas it used to be about living’ before the transition consumed them. Sally also acknowledged the impact on her husband who had even commented about having their daughter fostered due to her behaviour being ‘very nasty…very aggressive at times’.

For Louise, the transition negatively impacted her relationships beyond her immediate family due to avoiding disruptions to her son’s ‘time and routine’ and stability during the school term; this made it difficult to see friends and extended family. She was limited to seeing them in the school holidays when ‘he’s a bit more amenable’. It seemed unsurprising that the battle at school, alongside the transition’s effect at home, impacted on mothers’ emotional well-being.

#### Emotional impact on the mother

Two mothers described their underlying emotional and physical exhaustion due to the impact of transitioning. For example, Anna felt she was ‘running on empty most of the time’ and her ‘mind is constantly full’. Sally spoke about ‘sleepless nights’ and there being ‘nothing to give…that respite’ from the relentless responsibility of being the primary carer for her child. Indeed, Sally dreaded the prospect of year eight being equally demanding: ‘I don’t feel we could do another year like this year, because it’s been exhausting. Absolutely exhausting’, emphasising the all-consuming impact the transition had on her.

Each mother expressed unique concerns, with Anna, Juliet and Francesca specifically lacking ‘hope’ for the transition: ‘To be honest … I don’t know if I had any hopefulness’ (Juliet). Anna’s worry focussed on whether she had made the right choice of secondary school: ‘that was really hard to manage, because I kept thinking “is this the wrong decision, is this the wrong decision?”…there were so many concerns’; despite the soul searching she went on to emphasise the on-going nature of her trepidations stating she did ‘not think the future looks bright’.

Three mothers (Louise, Juliet, Diane) had doubts and anxieties about the transition, however, they recognised the importance of hiding these feelings from their child, remaining reassuring and supportive to maintain their child’s emotional well-being:

The more anxious you are as a parent regarding the transition, the more your child picks up on it. They’ve already decided that if you’re worried, they should be really worried. So even if I am anxious about it, I will do my best to not show that. (Louise)

All mothers indicated the practical impact of transition (the time commitment, discussions, regular communication with the school, attending structured transition programmes), whilst also managing autistic behaviours: ‘Even though you don’t let him have control, indirectly it ends up being like that. Everything is revolved around trying to make sure it runs smoothly’ (Louise). Given this meant Louise was no longer able to work, perhaps understandably she felt ‘bitter’ her life was ‘regimented’ due to new routines taking twice as long as they did at primary school. In contrast, Diane spoke positively that ‘it hasn’t held me back, in terms of my career’; instead, she changed job when her son started secondary school, giving her ‘more routine in…life’. This meant she could be ‘home every evening, which works better’. She did, however, feel the transition had been ‘going on for years’ as she was preparing her son for his ‘entrance test right from the beginning of year five’. Perhaps, to facilitate success, early transition preparation was essential for parents, families, and child.

## Discussion

This study aimed to explore parent’s experiences of their autistic child’s transition to secondary school and its impact on them as a family. Overarching themes highlighted the stressful and emotional nature of this transition. These findings were consistent with previous studies showing the emotional impact the transition had on a family with an autistic child (Makin *et al.* 2017). Importantly, the impact remained beyond the initial transition period and directly influenced other members of the family.

Stress was often underpinned by the necessity of mothers’ advocacy role to fight to obtain eligible services for their child (Ryan and Cole 2009; McMinn *et al.* 2018), with many mothers experiencing the transition period as a battle. For example, two mothers had to fight to get their children accepted by initially reluctant schools. Other battles were centred on the schools’ failure to implement agreed transition plans/support for their child. In some cases, eventually there were beneficial outcomes, with schools becoming receptive to implementing appropriate care. Conversely, when transition plans were not effectively implemented, parents were left feeling unsupported, alone, and alienated (Makin *et al.* 2017).

As well as taking on the burden of their child’s anxieties and behaviours, mothers highlighted how the transition affected their parenting role, extending to other aspects of their lives, with the whole family practically and emotionally impacted. While some mothers in the current study were able to maintain a healthy life balance; for others, new commitments from the transition reduced available time and energy to invest in family activities. This presented challenges to mothers’ own emotional well-being (Tobin *et al.* 2012). Siblings of autistic children have previously been identified as high risk for adjustment problems (Ross and Cuskelly 2006); our findings suggest they may be particularly vulnerable during their autistic siblings’ school transition. Although, Pavlopoulou *et al.* (2022) recently found non-autistic siblings to be most as risk from experiencing difficulties related to others’ lack of autism awareness and acceptance, rather than adjustment problems per se.

Importantly, all parents recognised the value of educating themselves on autism, accessing available support on offer and their rights. This supports previous work in which parents benefited from being armed with knowledge (Galpin *et al.* 2017) to ensure effective functioning within the system (Woodgate *et al.* 2008). Further, other parents of autistic children can offer mutual understanding of experiences and valuable guidance to navigate the system more effectively (Ryan and Cole 2009). This interaction reduced feelings of isolation and loneliness for two participants. However, some mothers had limited access to such opportunities and stated schools organising parent transition support groups would be useful. Online support communities have increasingly become an accessible way of gaining social, emotional, and informational support from peers (Soos *et al.* 2022), and the current findings suggest that these communities may be particularly useful for early and sustained transition planning.

The importance of communication between parents and schools for a successful transition is well documented (Makin *et al.* 2017, Peters and Brooks 2016, Bruck *et al.* 2022), with poor communication illustrated by the school’s failure to understand the importance of preparation and support for autistic individuals (Tobin *et al.* 2012). Common to every account in the current study was the mothers’ desire for schools to be transparent and open in communications with them; some mothers had more positive experiences with the school, involving collaborative practices, pre-transition programmes and efficient communication through a key point of contact providing regular feedback. Beyond this, given mothers’ knowledge of the psychological impact within the family from their child’s school transition, they wanted to be involved in equal decision-making processes with the school, in line with government guidance (Department for Education 2015).

Given the heterogeneity of autism symptom presentation, mothers wanted schools to value their maternal expertise and implement individualised guidance affording them peace of mind that their child was safe and progressing at school. As one mother highlighted, when teachers were aware of her son’s autistic behaviours and how to manage them, he was able to thrive. Previous studies have highlighted that parents have expectations of more individualised tailored support for the transition to secondary school (Parsons *et al.* 2009, Peters and Brooks 2016), with the uniqueness of each child’s needs preventing one-size-fits-all recommendations (Richter *et al.* 2020). Consequently, flexible and adaptable transition packages which provide individual tailored support options could be an effective strategy, enabling parents to adapt provision to their child’s specific needs. These adaptations may include reduced timetables if this supports the transition needs of the child, supported via parents sharing idiosyncratic information to improve communication about their child for the different stakeholders to use. SENCOs should liaise with staff on an ongoing basis about the strategies other teachers have found effective/ineffective with certain children when utilising adaptations permitted at an individualised level. Given some teachers may only see the child managing at school, maintaining an awareness of any broader impacts on the child by acknowledging parental accounts of altered behaviours and meltdowns at home, should be considered when making school-based adjustments.

The transition to secondary school has the potential to be a positive experience for autistic students, and success could validate their integration into mainstream schools, and later integration transitioning into adulthood. Importantly, specialised transition training and government support can improve transition outcomes for autistic children (Fontil *et al.* 2019) and adopting a more positive narrative around the primary-secondary transition could enhance children’s perceptions of it. For example, in the current study positive transition outcomes derived from schools running a transition group which supported children being introduced gradually into the school, as well as secondary school teachers visiting children in their primary school; such strategies enable children to have a safe, familiar face when they start secondary school. Importantly, these transition strategies could prove important for any child with a SEN diagnosis, and/or any child particularly worried about transitioning to a new school.

Instead of aiming for generalised claims, IPA aims for case-to-case transfer generalisation (Treharne and Riggs 2015), thus, this study did have a large enough sample for an IPA analysis to explore the data in depth (Smith *et al.* 2009). Nevertheless, as interviews often require extensive and intimate self-disclosure, it may encourage the recruitment of individuals with a specific interest in the topic than the general sample population (Robinson 2014), generating bias. For example, for two mothers, participation stemmed from the wish to help other parents avoid negative transition experiences. This is important as the study did not control for mothers’ expectations of the transition; research has shown those with more positive expectations are likely to be more open to transition experiences (Richter *et al.* 2020). Also, as only mothers were recruited, it is only their account forming understanding of others’ experiences, thus, exploring the voices of other family members, notably siblings, is an important avenue for future research. Finally, this study benefited from conducting interviews towards the end of the child’s transition year compared to those that have focused on the beginning phase of the transition to secondary school (e.g. Cremin *et al.* 2017), helping mothers to identify transition-based issues that only emerged later in the school year. As the transition period lasts longer than the first few weeks of the school year, future studies would benefit from follow-up with parents at regular intervals throughout school to understand the longer-term impact of the journey into secondary school.

The current study highlighted some of the concerns parents had about their families experience of the transition. Importantly, the parents also offered important practical implications for those who had more positive experiences. For example, important considerations to support the process of the transition included the desire for collaboration between parents and educators, on-going open communication and parental involvement in the decision-making process, the need for autism-specific individualized guidance. Moreover, there needs to be more focus from professionals and teachers on the support offered to siblings, which may include closely monitoring the sibling following this transition period but could also include opportunities for more one-to-one interactions or even sibling-led groups (Pavlopoulou *et al.* 2022). Importantly, there needs to be a safe space offered at school where the sibling feels able to share their feelings and request help as and when required.
